# Mechanical Characterization of Polysilicon MEMS: A Hybrid TMCMC/POD-Kriging Approach

**DOI:** 10.3390/s18041243

**Published:** 2018-04-17

**Authors:** Ramin Mirzazadeh, Saeed Eftekhar Azam, Stefano Mariani

**Affiliations:** 1 Department of Civil and Environmental Engineering, Politecnico di Milano, Piazza L. da Vinci 32, 20133 Milano, Italy; ramin.mirzazadeh@polimi.it; 2 Department of Civil Engineering, University of Nebraska-Lincoln, 2200 Vine St, Lincoln, NE 68503, USA; eftekhar-azam@unl.edu

**Keywords:** micro electro-mechanical systems (MEMS), uncertainty quantification, transitional Markov chain Monte Carlo (TMCMC) analysis, reduced-order modeling, polysilicon morphology, overetch

## Abstract

Microscale uncertainties related to the geometry and morphology of polycrystalline silicon films, constituting the movable structures of micro electro-mechanical systems (MEMS), were investigated through a joint numerical/experimental approach. An on-chip testing device was designed and fabricated to deform a compliant polysilicon beam. In previous studies, we showed that the scattering in the input–output characteristics of the device can be properly described only if statistical features related to the morphology of the columnar polysilicon film and to the etching process adopted to release the movable structure are taken into account. In this work, a high fidelity finite element model of the device was used to feed a transitional Markov chain Monte Carlo (TMCMC) algorithm for the estimation of the unknown parameters governing the aforementioned statistical features. To reduce the computational cost of the stochastic analysis, a synergy of proper orthogonal decomposition (POD) and kriging interpolation was adopted. Results are reported for a batch of nominally identical tested devices, in terms of measurement error-affected probability distributions of the overall Young’s modulus of the polysilicon film and of the overetch depth.

## 1. Introduction

By combining electronic and structural components, micro electro-mechanical systems (MEMS) have been a successful example of micro-technology surpassing conventional ones for a variety of devices like e.g., accelerometers, magnetometers, scanners, pressure sensors and gyroscopes [1]. However, the development of these devices involves relatively complicated steps, due to their small size scale. Testing and characterization are obviously necessary to predict the operational performance of a new device, optimize the micro-fabrication processes and develop applications [2,3]. Such tasks may become complex due to the presence of unknown mechanical or geometrical parameters, featuring large uncertainty levels at the microscale. The current miniaturization of the mechanical components, as needed for ever smaller devices, is enhancing the importance of such uncertainty issues in the fabrication process [4]. In fact, the small dimensions of miniaturized devices can go in the direction of micro-fabrication tolerances and of characteristic lengths linked to material heterogeneities. Therefore, improvements of our understanding of the involved sources of uncertainty can largely affect the success of newly designed devices to meet application constraints [5,6]. Even if the discussion to follow is focused on the operational conditions of the devices, when, from the mechanical perspective, no dissipation mechanisms take place in their movable structures, some reliability issues might be triggered by uncertainties, as linked to the propagation of microcracks in the polysilicon film [7,8,9].

Geometrical uncertainties, namely variations of the fabricated device geometry away from the designed layout, strongly depend at this length-scale on the fabrication process tolerances (see e.g., [10]). For instance, in [11,12], flexure width variations up to 0.4 μm and 0.7 μm were respectively reported. Fabrication inaccuracies can then result in a 10% variation of the geometry of conventional MEMS [10], and can consequently spoil or compromise the target performance. Variations in the material properties (like e.g., the Young’s modulus) can instead have intrinsic reasons, in addition to the dependency on the micro-fabrication process. This is especially the case for micro-sized components made of polycrystalline materials like polysilicon. As the dimensions of the components shrink, stochasticity of crystal orientation and grain morphology can lead to a scattered mechanical behavior of the whole component. This issue was reported numerically in [13,14] and experimentally in [15], for a decreasing material sample size.

A successful approach to assess the two aforementioned types of uncertainty has to be based on an experimental method and a relevant modelling/data reduction strategy. Due to the diversity of fabrication processes and MEMS applications, a unique approach to the mentioned assessment does not exist: a plethora of methods can be found in the literature to characterize the material at the microscale. The most common ones are based on tension and bending deformations of a specimen; a classification can be made depending on the test setup, on the sample preparation and on the actuation and read out methods. Reviews of these methods can be found, e.g., in [5,16,17,18]. Common approaches to characterize process variations at the microscale are instead based on surface inspections through optical or scanning electron microscopy [19,20], or laser interferometry [21]. These methods, even if characterized by a rather easy data reduction, are generally expensive and need the devices to be on open wafers, so that they are limited to pre-packaged testing. In [22], a testing platform using laser Doppler vibrometry was proposed to reduce the testing time, but still on unpackaged devices. An alternative approach was adopted in [11] to measure the capacitance change induced by an applied voltage, using standard electrical probing. This metrology is inexpensive and also applicable to packaged devices; nevertheless, due to the adopted data reduction method, it can be used only for measuring geometrical features.

In this paper, following our former works [23,24,25], a methodology is proposed to deal with the response of an on-chip testing device. The method is based upon standard electrical probing, and aims to stochastically characterize geometrical and material properties of polysilicon films through a flexible and powerful data reduction technique. The testing device here exploited, as introduced in [23], features a micro-cantilever film sample subjected to a bending deformation. Within a stochastic framework, the experimental results are interpreted by means of a transitional Markov chain Monte Carlo (TMCMC) analysis, to also account for the effects of measurement errors on the estimation of the unknown parameters ruling geometrical and material uncertainties. A high fidelity, finite element (FE) model of the testing device was adopted to properly represent the complexity of the system, potentially in terms of the polysilicon morphology too [25]. Since the handling of FE simulations is prohibitive in a TMCMC analysis, a parametric model order reduction method was proposed to reduce the computational effort, preserving the required accuracy of the solution. Such order reduction method is based on a hybrid use of proper orthogonal decomposition and kriging metamodeling [26,27].

For ten devices randomly chosen from the same wafer, the proposed approach has been used to quantitatively assess the effective stiffness of the micro-cantilever and the overetch depth, the latter being defined as a defect in the in-plane geometry of the movable structure caused by fluctuations of temperature and etchant concentration [28] that lead to a real geometry of the device different from the target one. Both polysilicon elastic properties and overetch depth were assumed isotropic in the plane of motion, not only in terms of their most probable values but also through their scattering around the mean.

The remainder of this paper is organized as follows. In Section 2, some details of the on-chip testing device are provided; the measurements collected for a batch of nominally identical specimens are also discussed. In Section 3, the numerical procedure adopted to model the coupled electro-mechanical response of the device is detailed, focusing on the methodology for reduced-order modeling based on proper orthogonal decomposition and kriging interpolation in the domain of variation for the parameters to be identified. Moving to the identification procedure through a Bayesian approach, Section 4 gathers the major details of the adopted TMCMC methodology (while additional technical details are given in the Appendix A). Results of parameter estimation are then collected and discussed in Section 5. Finally, some concluding remarks and comments on future activities are provided in Section 6.

## 2. On-Chip Testing Device: Experimental Data and Relevant Scattering

**Figure 1:**
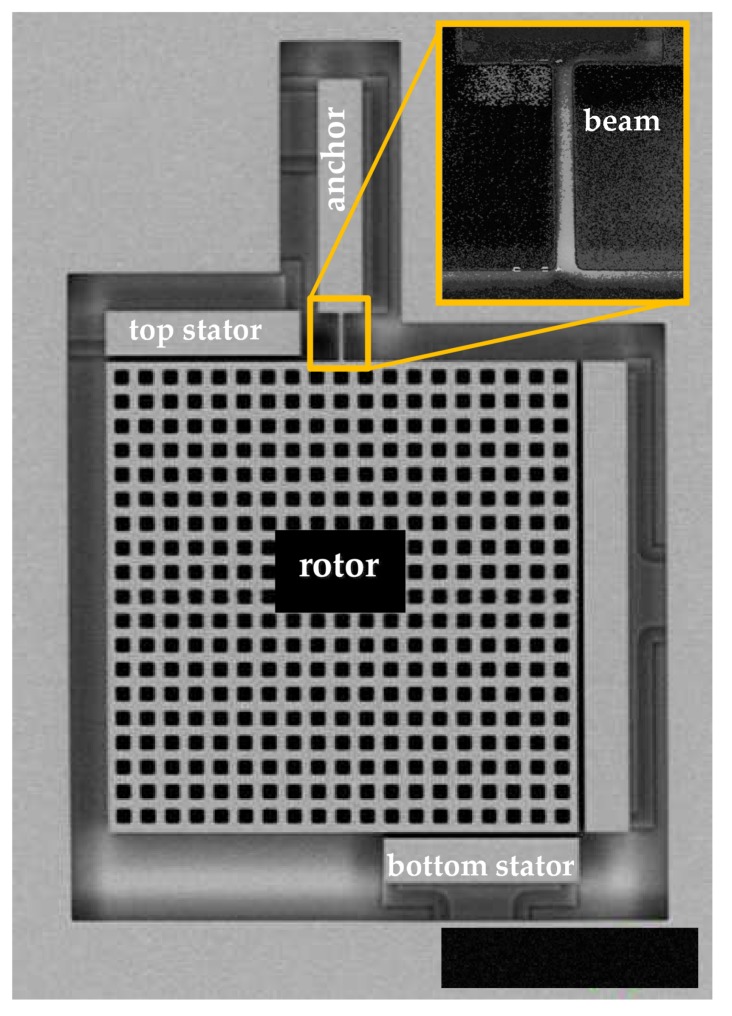
SEM picture of the on-chip testing device.

The on-chip testing device proposed in [23] for the estimation of micromechanics-induced uncertainties in polysilicon MEMS, was fabricated through the ThELMA process by STMicroelectronics, Geneva, Switzerland (see e.g., [29]). An SEM picture of the mechanical part of the device is shown in Figure 1: the massive perforated plate (denoted as rotor) and the stators surrounding it were designed to deform, through an electrostatic actuation, the slender cantilever beam shown in the close-up. This beam or spring is connected to the substrate on its top side and to the rotor on its bottom side. Such a statistically determinate mechanical scheme of the movable structure was devised to reduce to a minimum the effects of residual stresses on the measured response to the actuation. Although a slight in-plane offset of the rotor can be induced by gradients of the residual stress field inside the polysilicon film, such effect can be considered negligible (see [24,25]). The target geometrical characteristics of the tested devices are summarized in Table 1. The structure has been packaged at a pressure of 0.35 mbar, so that the working conditions are close to those of similar commercial devices.

Under quasi-static loading conditions, with the rotor electrically grounded and a bias voltage applied to the stators, attractive forces easily reduce the gap at the capacitors due to the slenderness of the beam. Because of the symmetric position of the top and bottom stators about the longitudinal axis of the beam, a pure bending is induced in the cantilever and the rotor gets displaced (mainly rotated) as a rigid body. For capped devices, the voltage-induced change of the mechanical configuration could not be assessed through visual inspection; the change of the capacitance at the same capacitors where the voltage is applied was then measured to sense the deformation of the beam.

Due to the presence of the lateral stator shown in Figure 1, different deformation modes can be induced in the beam. In this work, only the aforementioned purely bending one is investigated, to focus on the statistics and dispersion of the results induced by micromechanical features and by the measurement noise. Additional details on this device and on its response to the actuation can be found in [23,24,25,30,31,32].

**Figure 2:**
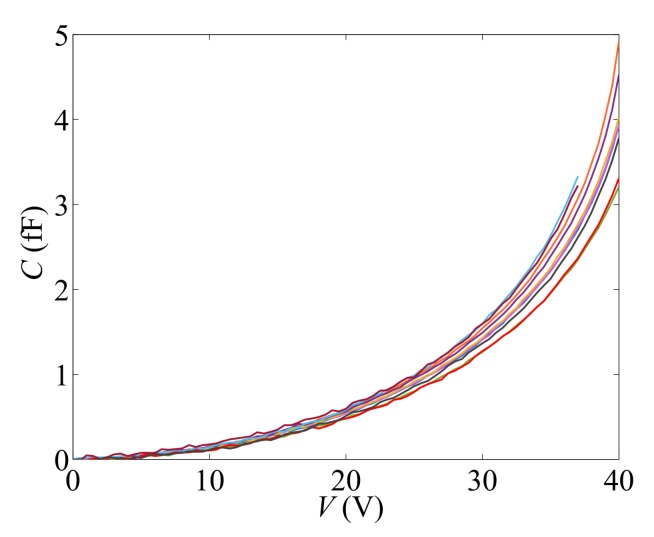
Experimentally measured capacitance change *C* vs. applied voltage *V*.

Since the cantilever was purposely designed as thin as possible, in compliance with the micro-fabrication constraints, a remarkable spreading in the mechanical response of nominally identical devices was already reported and is studied here. Ten specimens were taken from a single wafer, and their response measured in terms of capacitance change *C* against the applied voltage *V*: results are all collected in Figure 2. The different colors used in this figure to distinguish each single response from all the others will be exploited in Section 5 to correlate the stiff/complaint response in this *C*–*V* plane with the final estimates of the unknown parameters characterizing the devices (see below).

The goal of the experimental campaign was not an assessment of the pull-in instability; in fact, in [23], we showed that a one-to-one relationship does not exist between the pull-in voltage and the micro-fabrication uncertainties. Accordingly, the devices that displayed a more compliant response were actuated up to lower voltage levels, so as to avoid any damage and allow repeated testing. The scattering shown in the graph, which was reported in [25] to depend on the properties of the devices and not on measurement errors, is therefore directly related to uncertainties at the microscale. Among them, one major source is induced by the polysilicon film morphology at the beam level, which is different for each device. Such effect was shown in [23,30] to give rise to variations of the measured (effective) Young’s modulus of the microcantilever, on the order of ±5% with respect to the reference isotropic value [14]. Another uncertainty source is related to the patterning/etching stages of the production process, which do not provide a uniform geometry of the moving structure over the whole wafer (see, e.g., [12]). Accordingly, the overetch defect [32], which modifies the in-plane dimensions of the device components with respect to the target ones, needs to be considered too.

In former works [23,32], it was already pointed out that estimating the effects of these two main sources of uncertainty from the overall device response is not a trivial task. As already stated, data concerning the pull-in voltage alone are not enough to micromechanically characterize each single device, due to its nonlinear response in terms of the *C*–*V* plot and to the intricate, combined effects of film morphology and overetch depth. Within a fully stochastic frame, the evolution of the system response under an increasing applied voltage is accounted for here, along with the measurement noise.

To slightly simplify the problem, in compliance with previous studies, the microstructural uncertainties are assumed to depend on two unknown parameters only: the overetch *O*, assumed to be homogeneous and direction-independent for each single device; and the effective Young’s modulus *E* of the polysilicon beam. Variations of *O* affect the thickness and the length of the beam, and the gap at capacitors in the initial configuration; variations of *E* affect instead the stiffness of the beam only.

## 3. Reduced-Order Modelling

To identify the characteristic values and the dispersion of parameters *O* and *E*, as featured by the batch of devices that furnished the responses gathered in Figure 2, an inverse problem has to be solved. By comparing the experimental data with the results of micromechanics-informed numerical simulations, the mentioned stochastic properties of the parameters can be identified, also handling the measurement error. Such characterization of the polysilicon film will be therefore richer in the content of information if compared to those provided in [23,24], for which the ill-conditioning of the inverse problem [33] would have potentially affected the solution.

As will be detailed in Section 4, a TMCMC method for Bayesian parameter estimation was adopted. This type of investigation requires a large number of simulations to properly catch all the effects of the parameters to be identified on the nonlinear transition between the input parameter (the applied voltage *V*) and the output one (the measured capacitance change *C*) (see [34]).

Each high-fidelity, coupled electro-mechanical simulation is based on a finite element (FE) discretization of the mechanical and electrical domains. Although the type of actuation described in Section 2 gives rise to a deformation of the movable structure that can be handled through simplified, beam bending-like analytical approaches like those explored in [23,31], the FE analyses can provide a more general framework and account for effects disregarded in the analytical models (like e.g., those linked to anchor compliance, re-entrant corners close to the final cross-sections of the cantilever, and fringe fields in the electrical domain) (see also [25]). Handling such FE analyses within an inverse analysis turns out to be computationally prohibitive, and so the overall computational burden of the procedure must be reduced.

To preserve as much as possible the accuracy of the solutions, we discuss next a methodology for model order reduction, which is based on a set of system responses (also called snapshots) at varying values of the unknown parameters to be estimated. As the investigation aims to get insights into the (nonlinear) effects of those parameters on the *C*–*V* response of the devices, this methodology is referred to as parametric model order reduction [35]. Algorithmically, the method is based on a coupling of proper orthogonal decomposition (POD) and kriging interpolation, to build a surrogate of the initial full-order FE model. POD is used to quantify the correlation among the snapshots, and then set the subspace onto which the full-order model response can be projected; kriging is used to predict (and not compute) the system response for any values of the unknown parameters different from those adopted to obtain the snapshots.

Parametric model order reduction strategies similar to the one adopted here were already proposed for nonlinear problems, even within a multi-scale framework, in e.g., [36]. As long as the response of the system evolves smoothly in the inspected domain of variation of the parameters, wherein the interpolation is introduced, the aforementioned strategies can work quite satisfactorily (see also [37,38]); whenever the response varies instead non-smoothly, for instance as due to crack evolution, the applicability of the methodology has to be proven.

### 3.1. Proper Orthogonal Decomposition

We briefly review first the key features of POD for building a model-specific optimal subspace from the full-order FE model, based on an ensemble of responses. A general and synthetic introduction to POD for parametric reduced-order modelling can be found in [39] (see also [40,41]).

We start by considering the case of a single FE analysis, at assigned values of *O* and *E* collected in the vector x of unknowns. Later, we will extend this solution to the case of multiple x values, in order to allow for parametric dependence in the solution.

Let $c\in R^{m}$ be a vector representing the device response, measured at a progressively increasing value of the input actuation. Here, R is the set of real numbers, and *m* denotes the dimension of c. For the present case, c collects the values of the capacitance change *C* linked to a set of values of the applied voltage *V*. Even though the response of each device smoothly evolves with *V* (see Figure 2), vector c is designed to gather a set of values of *C* to exploit redundancy and to also reduce the effect of measurement noise on the final estimates of *O* and *E*. The vector c can be represented as a superposition of orthonormal bases $\phi_{i}$, i=1,2…,m, according to:

(1) $$c=\sum_{i=1}^{m}\phi_{i}a_{i}=\Phi a,$$

where $a_{i}$ are the expansion coefficients or, in other words, the amplitudes of the bases $\phi_{i}$ in the solution. In compliance with the notation of Equation (1), the aforementioned coefficients are gathered in the column vector a, while matrix $\Phi =[\phi_{1},\phi_{2},\ldots ,\phi_{m}]$ collects all the bases, or proper orthogonal modes (POMs) as columns. By truncating the expansion in Equation (1) according to:

(2) $$c\approx \hat{c}=\sum_{i=1}^{q}\phi_{i}{\hat{a}}_{i}=\hat{\Phi }\hat{a}$$

with q<m, a reduced-order representation of the original model is obtained. The matrix $\hat{\Phi }$ thus consists of the first *q* bases only, whereas the vector $\hat{a}$ collects the corresponding weighting coefficients, which are different from those in a to compensate for the truncation errors. The computational gain when moving from Equation (1) to Equation (2) is related to the ratio q/m between the degrees of freedom of the reduced-order and of the full-order models. The aim of the POD-based reduced order modeling procedure is to provide, at assigned *q*, an optimal set of bases able to minimize the truncation error in Equation (2); alternatively, POD can provide the value of *q* to guarantee a requested accuracy of the order reduction [42]. The snapshot version of POD, see [39], can address both of the aforementioned problems at the same time, setting *q* to attain the requested accuracy and providing the $\hat{\Phi }$ matrix.

To move now to the solution of the parametric reduced-order model problem, we consider *n* different systems, each one featuring its own values of the unknown parameters *O* and *E*. All the responses are collected in the snapshot matrix C, so that the representation can be written as:

(3) $$C=\left[c_{1},c_{2},\ldots ,c_{n}\right]\approx \hat{\Phi }\left[{\hat{a}}_{1},{\hat{a}}_{2},\ldots ,{\hat{a}}_{n}\right]=\hat{\Phi }\hat{A}.$$

Matrix C can be factorized through a singular value decomposition (SVD), to provide [40,43]:

(4) $$C=L\Lambda R^{T},$$

where L is an orthonormal matrix, whose columns are the left singular vectors of C; Λ is a pseudo-diagonal and semi-positive definite matrix, whose pivotal entries $\lambda_{ii}$ are the singular values of C; R is an orthonormal matrix, whose columns are the right singular vectors of C. Granted that the singular values of C are sorted in a decreasing order, Equation (4) provides the sought basis set via L. The order *q* of the reduced-order model is finally set by fulfilling:

(5) $$q=\underset{\nu }{\inf}\frac{\sum_{i=1}^{\nu }\lambda_{ii}^{2}}{\sum_{i=1}^{m}\lambda_{ii}^{2}}\geq 1-\varrho ,$$

where ϱ is the assigned error threshold (typically small, i.e., ϱ≅1−5%).

Being related to the snapshots in C, the accuracy provided by Equation (5) holds true only for the handled values of the unknowns in $x_{1}$, $x_{2}$, …, $x_{n}$. To ensure the capability to approximate the system response also for values of x different from those adopted to obtain the snapshots, so for any arbitrary combination of the ruling parameters in their domain of variation, an interpolation needs to be introduced. By assuming that $\hat{\Phi }$ is not affected by x, we state:

(6) $$\hat{c}\left(x\right)=\hat{\Phi }\hat{a}\left(x\right),$$

where the approximate response $\hat{c}\left(x\right)$ and the weighting functions $\hat{a}\left(x\right)$ depend on x through an interpolation to build around the coefficients stored in matrix $\hat{A}$.

In Equation (6), the reduced-order basis matrix $\hat{\Phi }$ is not supposed to vary significantly with x, as the snapshots collected in C are assumed to provide physically-sound and accurate information on the actual system behavior, independently of x. The combination coefficients $\hat{a}$ instead depend on x, as different parameter values may selectively increase the importance of each mode in $\hat{\Phi }$. Since the system response is supposed to smoothly depend on *O* and *E* up to pull-in, a smooth interpolation $\hat{a}\left(x\right)$ is looked for within their ranges of variation. Accordingly, system nonlinearities linked to the effect of x on the *C*–*V* curve are covered by the interpolation functions $\hat{a}\left(x\right)$; nonlinearities related instead to the *C*–*V* plot itself at assigned x, are (approximately) accounted for when building the modes retained in $\hat{\Phi }$.

### 3.2. Kriging Interpolation

To build the functions $\hat{a}\left(x\right)$ or an approximation for them, kriging is discussed next. This methodology already showed a high performance, in terms of accuracy and robustness, when adopted to obtain surrogates for nonlinear systems [44,45]. In what follows, we denote with $\hat{\alpha }\left(x\right)$ the obtained approximating functions, to distinguish them from the correct ones $\hat{a}\left(x\right)$; since the sought smoothing is aimed at minimizing the interpolation errors within the whole domain of variation of the parameters, the computed solutions may not even match some of those collected by the snapshots. Kriging looks particularly attractive for the present investigation as it provides a probabilistic framework to build $\hat{\alpha }\left(x\right)$, which obviously depends on the training data obtained with the full-order simulator.

As the standard goal of kriging is to furnish an estimate of a scalar function, we build an individual emulator for each entry of the interpolation vector. Hence, ${\hat{\alpha }}_{i}\left(x\right)$, i=1,2,…,q, is assumed to be a sample path from a Gaussian process, which needs to be characterized in terms of its mean and autocovariance functions. To get these features, the available coefficients of matrix $\hat{A}$ (see Equation (3)) are exploited. For ease of notation, the index *i* is dropped in what follows and reference is made to the scalar function $\hat{\alpha }\left(x\right)$; according to the preceding discussion, the procedure reported in what follows has to be repeated *q* times, handling the interpolation functions one-by-one.

With universal kriging, it is assumed that the Gaussian process under investigation is centered around a regression model, known as a trend [46], in compliance with:

(7) $$\hat{\alpha }\left(x\right)=\sum_{k=1}^{s}\beta_{k}f_{k}\left(x\right)+Z\left(x\right)=f^{T}\left(x\right)\beta +Z\left(x\right),$$

where $f^{T}\left(x\right)=\left\{f_{1}\left(x\right),\ldots ,f_{s}\left(x\right)\right\}$ are the regression functions, and $\beta ={\left\{\beta_{1},\ldots ,\beta_{s}\right\}}^{T}$ are the corresponding regression coefficients. The number of regression functions is assumed s≤n (where *n* is the number of observations), so as to avoid managing an under-determined problem. Function Z(x) has zero mean, and a variance $\sigma_{\alpha }^{2}R(|x-x^{\prime }\left|,\xi \right)$ depending, besides $\sigma_{\alpha }^{2}$ and ξ, on the distance between the two points x and $x^{\prime }$ in the parameters space. Since the correct representation of the data critically depends on the type of correlation function *R*, parameters ξ, β and $\sigma_{\alpha }^{2}$ have to be determined on the basis of the chosen correlation and regression functions. Various types of functions are available in the literature (see [47]): the anisotropic generalized exponential model is the common choice for the correlation function [48], and low order polynomials of x are usually adopted for the regression model [46].

The vector ${\hat{a}}^{*}$, representing the responses computed through the simulator (and here meant as the row of matrix $\hat{A}$ in Equation (3) corresponding to the function α(x) currently handled), and the relevant emulator prediction $\hat{\alpha }\left(x\right)$, are all assumed to be normally distributed and then written as [49]:

(8) $$\left\{\begin{array}{c} {\hat{a}}^{*}\\ \hat{\alpha }\left(x\right) \end{array}\right\}∼N\left(\left\{\begin{array}{c} F\beta \\ f^{T}\left(x\right)\beta , \end{array}\right\},\sigma_{\alpha }^{2}\left\{\begin{array}{cc} R & r(x)\\ r{\left(x\right)}^{T} & 1 \end{array}\right\}\right),$$

where entries of F are computed as $F_{jk}=f_{k}\left(x_{j}\right)$, j=1,…,n, k=1,…,s; entries of the correlation matrix R are instead computed as $R_{ij}=R\left(x_{i},x_{j};\xi \right)$, i,j=1,…,n; vector r(x) of cross-correlations between the observation point $x_{j}$ and the prediction point x, is provided by $r_{j}\left(x\right)=R\left(x,x_{j};\xi \right)$, j=1,…,n.

The mean and variance of the conditional distribution of $\tilde{\alpha }\left(x\right)=\hat{\alpha }\left(x\right)|{\hat{a}}^{*}$, also known as mean and variance of the kriging predictor, are Gaussian as well and are respectively given by, see [46]:

(9) $$\mu_{\tilde{\alpha }}\left(x\right)=f^{T}\left(x\right)\tilde{\beta }+r{\left(x\right)}^{T}R^{-1}\left({\hat{a}}^{*}-F,\tilde{\beta }\right)$$

(10) $$\sigma_{\tilde{\alpha }}^{2}\left(x\right)=\sigma_{\alpha }^{2}\left(1-r{\left(x\right)}^{T}R^{-1}r\left(x\right)+p{\left(x\right)}^{T}{\left(F^{T}R^{-1}F\right)}^{-1}l\left(x\right)\right),$$

where:

(11) $$l\left(x\right)=F^{T}R^{-1}r\left(x\right)-f\left(x\right),$$

(12) $$\tilde{\beta }={\left(F^{T}R^{-1}F\right)}^{-1}F^{T}R^{-1}{\hat{a}}^{*}.$$

In the equations above here, $\tilde{\beta }$ is the generalized least squares solution (with respect to R) of the regression problem:

(13) $$F\tilde{\beta }≃{\hat{a}}^{*}.$$

Additional technical details of kriging were gathered in [30].

In practical terms, the regression and correlation models need to be selected first, and then kriging parameters can be determined via the provided statistical inference technique. Open software programs are available to obtain $\mu_{\tilde{\alpha }}$ and $\sigma_{\tilde{\alpha }}^{2}$, like e.g., the Matlab toolbox DACE [50] adopted in this work; in it, the built-in anisotropic generalized exponential correlation model and constant regression functions were used.

As far as the computational costs of kriging interpolation are concerned, it is worth noting that the procedure is used to derive metamodels for the weighting coefficients of the POMs, rather than directly for the response of the FE model (i.e., for data points in the *C*–*V* curves). Computational resources are therefore saved as kriging models that have to be built as many times as the number *q* of POMs retained in the reduced-order model, which are typically a few (on the order of q=2–4 for many applications, see also [43,51]), rather than as the number of the data points to define the *C*–*V* curves, which are instead many more to properly catch the nonlinear response of the device. This hybrid use of POD and kriging for model order reduction can be accordingly termed POD-kriging (see [26]).

**Figure 3:**
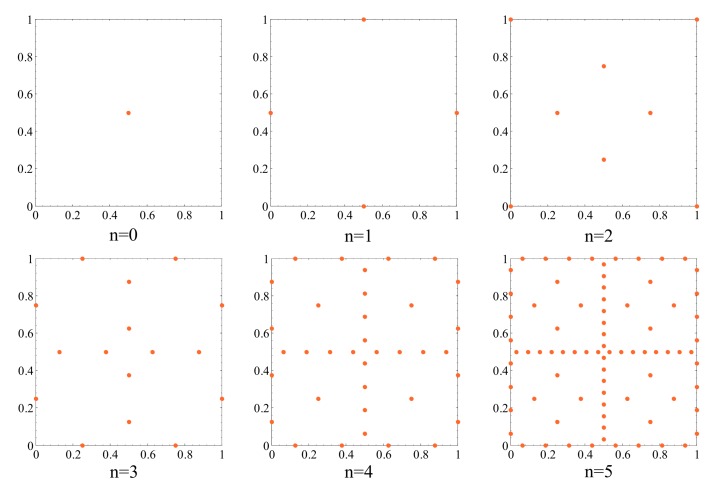
Illustration of the Clenshaw–Curtis sparse grid sampling point locations for an exemplary two-dimensional problem, at varying degree *d* of the interpolation.

To build such POD-kriging model, a set of simulations for given values of the parameter vector x is necessary; this step can be regarded as a sampling within the parameters space. Several sampling methods are available in the literature, such as Monte Carlo [52], Latin Hypercube [53], Halton [54], Sobol [55] and greedy [56] ones. In this work, sampling was based on the sparse grid Smolyak algorithm [57], which had been formerly adopted for kriging sampling in [58,59]. The most important feature of this technique is rooted in its hierarchical structure [60]: new sampling points, required if the surrogate does not attain a required level of accuracy, are plugged into the current set without any rearrangement of the already handled ones. This enriching stage of the surrogate becomes necessary whenever the initial set of the sampling points does not prove adequate to account for all the effects of model parameters on the POM mixture. Among the different sparse grid sampling procedures, the Clenshaw–Curtis one [59,61,62] was used to construct the POD-kriging model. The relevant hierarchical sequence of the sampling points within an exemplary (dimensionless) two-dimensional domain is sketched in Figure 3, at changing degree of interpolation: it is clearly shown that, by increasing the order *d*, the whole domain of variation of model parameters gets more densely inspected. The relevant number of sampling points to deploy at varying degree *d* is collected in Table 2, in order to also show that the adopted sampling method allows not to exponentially increase with *d*, so, with the accuracy of the reduced-order model, the computational burned related to the construction of the POD-kriging model.

## 4. Transitional Markov Chain Monte Carlo Method for Bayesian Parameter Estimation

The reduced-order modelling technique described in Section 3 was adopted to reduce a priori the computational burden of each analysis to carry out within an (offline) Bayesian parameter identification procedure, based on a TMCMC simulation [63]. The TMCMC method is one of the most advanced ones to deal with the optimization of relatively high-dimensional stochastic systems, and to sample complex probability density functions (PDFs) [64]. Within such framework, the unknown model parameters are viewed as random variables described by the relevant PDFs; this standpoint varies from deterministic approaches, dealing instead with the unknown parameters as deterministic variables, and generally failing to address the ill-posedness of the problem when noisy data and modeling inaccuracies are involved.

Within the proposed probabilistic inverse analysis, for each device, a population of possible solutions (in terms of unknown parameters x) is generated and then evolved according to the information collected through the *C*–*V* plot; the values providing the best fit to the experimental points are finally selected as the solution of the identification procedure. If $C_{exp}$ represents the system observations, namely the values of the capacitance change measured at varying actuation voltage, by applying the Bayes’ rule [65], one can sequentially update the probability of x conditioned on $C_{exp}$ through:

(14) $$p\left(x|C_{exp},M\right)\propto p\left(C_{exp}|x,M\right)p\left(x,M\right),$$

where p(x,M) represents the prior PDF of x, which accounts for the knowledge of the parameters before accounting for the observations; $p(C_{exp}|x,M)$ is the likelihood, which describes the probability of witnessing the data $C_{exp}$, given the parameters x in the model M. The proportionality factor, implicitly linking terms on the left- and right-hand sides of Equation (14), stands for the evidence of the model M to represent the measurements [34]. Within each successive iteration of the (reduced-order) model updating technique, Equation (14) converts the prior knowledge p(x,M) into the posterior density $p(x|C_{exp},M)$, having observed the data in $C_{exp}$.

In relation to this Bayesian framework, two important issues to address are the type of the likelihood, and how to properly reconstruct the posterior PDF. By relying on the central limit theorem, the likelihood can be assumed Gaussian; interested readers can find a detailed discussion on the choice of the likelihood function in [66]. The posterior distribution can be instead reconstructed using stochastic simulation methods, able to generate samples distributed according to such PDF. MCMC algorithms are typically recommended for this type of Bayesian simulations (see e.g., [34]): they involve a random walk (i.e., a succession of random steps) through the PDF, favoring values with higher probability. By repeating these random walks, every point in the parameters space is hit with a frequency proportional to its probability, so that the stationary distribution of the Markov chains is equal to (or at least it is proportional to) the target PDF.

Within this class of algorithms, the TMCMC relies on sampling from a sequence of Γ non-normalized intermediate PDFs $p_{\gamma }\left(x\right)$ according to, see [63]:

(15) $$\begin{array}{c} p_{\gamma }\left(x\right)=p{\left(C_{exp}|x,M\right)}^{\eta_{\gamma }}p\left(x,M\right),\;\gamma =0,\ldots ,\Gamma , \end{array}$$

where the tempering parameters $\eta_{\gamma }$ monotonically increase with γ (so that $0=\eta_{0}<\eta_{1}<\ldots <\eta_{\Gamma }=1$), to conceptually resemble simulated annealing [67]. By managing the experimental measurements collected in $C_{exp}$, the TMCMC adopts these tempering parameters to dilute the effect of data being used in the Γ-1 sampling sequences.

We assume now that $N_{s}$ samples $x_{k}^{\left(\gamma \right)}$, $k=1,\ldots ,N_{s}$ are taken from each intermediate PDF $p_{\gamma }\left(x\right)$; accordingly, this is done also at the initiation stage handling p(x,M). A technique similar to importance sampling is then used to generate the distribution $p_{\gamma +1}\left(x\right)$, where the importance weights are given by:

(16) $$w\left(x_{k}^{\left(\gamma \right)}\right)=\frac{p_{\gamma +1}\left(x_{k}^{\left(\gamma \right)}\right)}{p_{\gamma }\left(x_{k}^{\left(\gamma \right)}\right)}=p{\left(C_{exp}|x_{k}^{\left(\gamma \right)},M\right)}^{\eta_{\gamma +1}-\eta_{\gamma }}$$

and next normalized through:

(17) $$\tilde{w}\left(x_{k}^{\left(\gamma \right)}\right)=\frac{w(x_{k}^{\left(\gamma \right)})}{\sum_{k=1}^{N_{s}}w\left(x_{k}^{\left(\gamma \right)}\right)}.$$

By adopting a standard importance sampling procedure, a resampling stage is necessary to move the samples with the highest normalized weights onto the next intermediate distribution. This way, the method would soon face the well-known sample degeneracy problem [68]. To avoid such issue, the TMCMC procedure handles each resampled value $x_{k}^{\left(\gamma \right)}$ as the starting point of a Markov chain, which evolves in accordance with the Metropolis–Hastings algorithm [69], and along which each normalized importance weight determines the relevant chain length. A major feature of the present method is that alternate Markov chains, growing towards the high probability regions of the posterior, allow for also reconstructing multimodal PDFs. The algorithmic details of the TMCMC method used in this work is summarized in the Appendix A (see also [63]).

## 5. Results: Parameter Identification via POD-Kriging and TMCMC Analysis

Referring to the micromechanically governed problem introduced in Section 3, the high-fidelity FE model driving the parameter identification procedure was developed according to [30] (see also [25]). To study the in-plane motion of the rotor induced by the top and bottom stators, a two-dimensional discretization was adopted. The movable structure was modeled with quadratic triangular elements with displacement degrees of freedom only, whereas the gaps at capacitors were discretized with linear elements featuring both displacement and voltage degrees of freedom. To describe the configuration changes induced by the applied voltage, which leads to a consistent narrowing of the aforementioned gaps, the number of elements across the thickness of the electrical domains was selected to provide accurate and mesh-independent results, up to situations close to pull-in. Concerning the mechanical domain, to account for the possible stress intensification close to the re-entrant corners at the final cross-sections of the beam, the mesh was locally refined. This overall resulted into a large number of degrees of freedom, amounting to around 250,000, somehow depending on the overetch value that affects the structural geometry.

**Figure 4:**
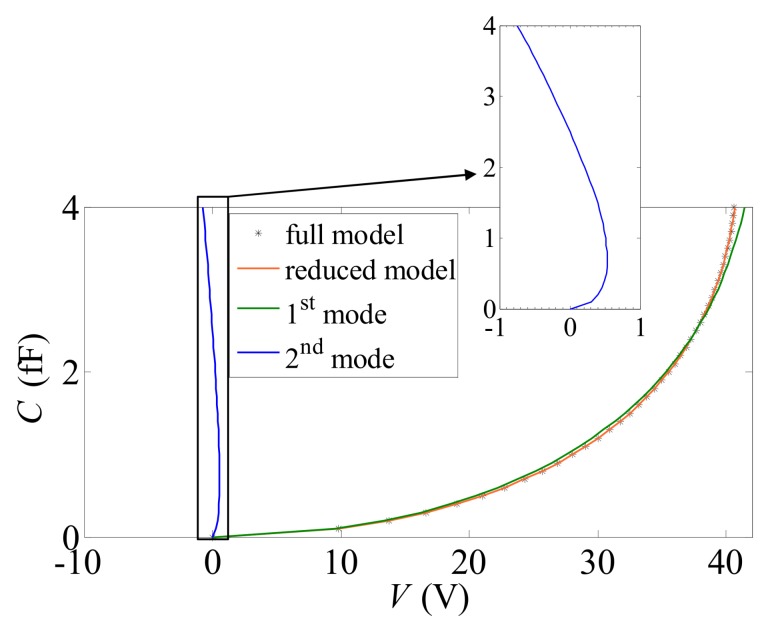
Comparison between full-order and reduced-order (featuring q=2) responses for a device characterized by *O* = 0.15 μm and *E* = 169 GPa, and representation of the two POMs retained to feed the kriging interpolation.

By adopting the proposed POD-kriging method, the computing time for the whole analysis up to pull-in, has been decreased from 3600 s to a few milliseconds on a personal computer featuring an Intel^®^Core^TM^i7 3.00 GHz as processor and Windows 10 as operating system. The ensemble of snapshots used for POD-kriging surrogate modeling was generated using the full-order model of the device, at varying values of *O* and *E* within the domain: O∈[−0.150.15] μm; E∈[130169] GPa. Values of the two parameters for the training analyses were selected in accordance with the Clenshaw–Curtis grid. A set of 65 FE simulations was run (see Table 2), adopting an interpolation of degree d=4 in the domain of variation of the parameters (see Figure 3), and leading to an error ϱ<1% in the POD truncation by keeping the first two POMs only in the reduced-order model (i.e., q=2). The shapes of these POMs are illustrated in Figure 4, where the *C*–*V* curve relevant to a device featuring *O* = 0.15 μm and *E* = 169 GPa and its POD representation are compared. To assess the contribution to the overall response provided by each mode, the two POMs are also reported separately. While the amplification of each POM represented in Figure 4 is specific for the aforementioned values of the unknown parameters, a similar trend was shown by all the other simulations.

**Figure 5:**
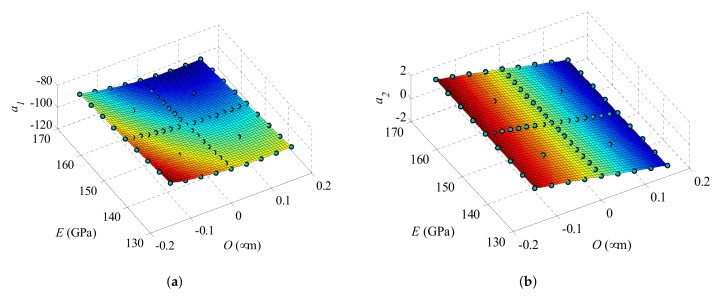
Dependence on *O* and *E* of the two interpolation functions (**a**) ${\hat{\alpha }}_{1}$ and (**b**) ${\hat{\alpha }}_{2}$ used to weight the effects of the POMs retained in the surrogate model.

The kriging interpolation functions $\hat{\alpha }\left(x\right)$ (see Section 3.2) are reported in Figure 5 over the whole domain of variation introduced before. It is shown that ${\hat{\alpha }}_{1}$ nonlinearly varies with *O* and *E*; ${\hat{\alpha }}_{2}$ instead varies almost linearly with *O*, being almost independent of *E*. This latter feature, together with the difference in the amplitudes of the two interpolation functions in the studied range, allow stating that, in case of variation of the Young’s modulus *E* only, the *C*–*V* curve can be fairly well presented using one POM only, whose amplitude nonlinearly depends on the value of *E* itself.

To estimate the specimen-dependent unknown values of *O* and *E*, the TMCMC algorithm was run using 10,000 samples for each stage. Thanks to the low computational burden associated with the POD-kriging model, the whole parameter identification procedure took up to 9 min for the analysis of each device on the same personal computer mentioned above. In the analysis, the measurement noise was assumed to be an additive white Gaussian process, with zero mean and standard deviation $\sigma_{m}=10^{-3}$ fF, in accordance with the experimental details provided in [25]. Since no prior knowledge on the unknown parameters was available, their initial distribution in the domain of variation was considered uniform.

**Figure 6:**
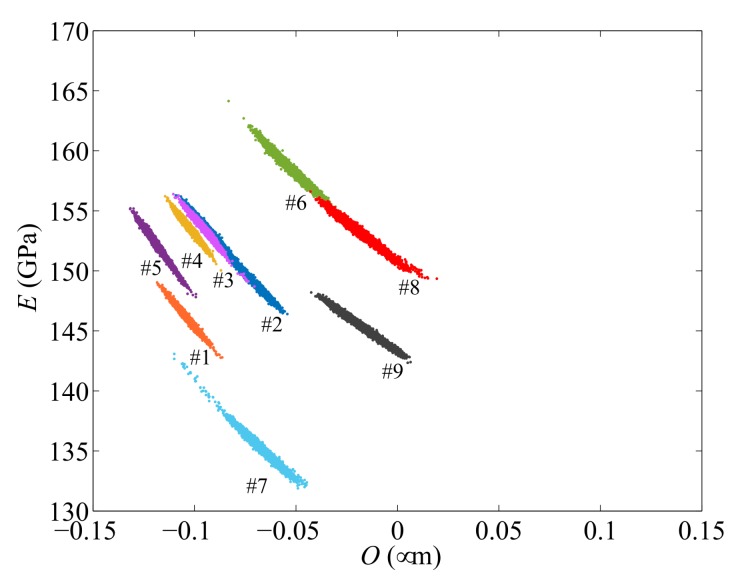
Final posterior distributions of the TMCMC samples in the *O*–*E* domain, as obtained for nine of the tested devices.

The results of the TMCMC analyses are plotted in Figure 6, in terms of the spreading of the samples at the end of the identification procedure. Results are relevant to nine specimens, out of the ten experimentally tested; those relevant to the last specimen will be discussed later in this section, and they are not included here as the procedure did not provide convergence towards feasible values of the two unknown parameters, at least within the considered domain of variation. The same color coding adopted in Figure 2 is managed here, as already commented on, to provide a visual correlation between the *C*–*V* response and the obtained estimates of *O* and *E*. Outcomes show that the values of overetch and Young’s modulus vary from one specimen to another: for all the cases, *O* turns out to be bounded between −0.1 μm and 0, while *E* significantly varies between 130 GPa and 160 GPa. Sample distributions also testify to a negative correlation between *O* and *E*: larger values of *O* correspond to lower values of *E*, and vice versa. Such correlation between the two unknown parameters and the dispersion of the samples corresponding to each device reveal a potential ill-posedness of the problem due to the existence of locally flat areas of the objective function implicitly handled in the identification procedure. Dealing with such objective function by means of a deterministic approach (see e.g., [24]) often fails to provide a reliable estimation of *O* and *E* for all the specimens.

**Figure 7:**
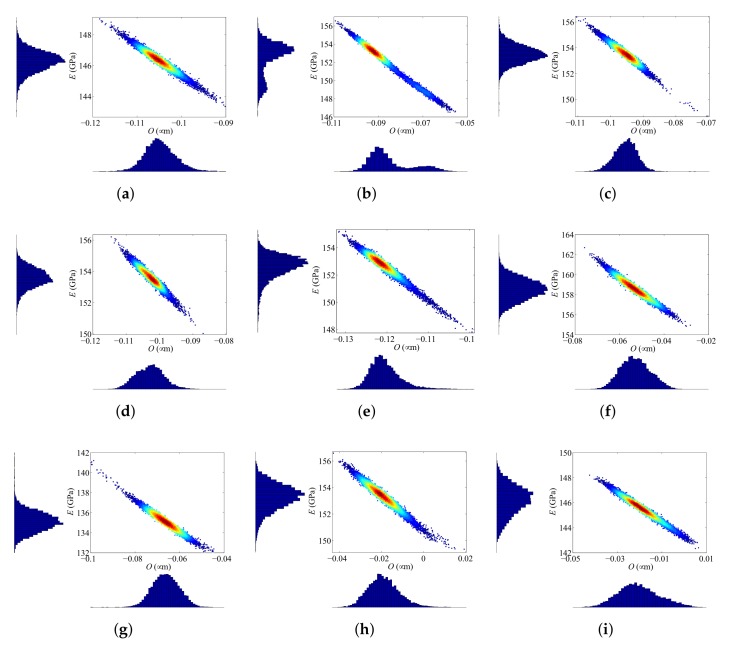
Scattering of the TMCMC samples, and corresponding histograms (reported on the bottom and left sides of each plot) of the model parameter distributions for devices (**a**) #1 to (**i**) #9.

The final specimen-dependent estimates can be better assessed by looking at the distributions of the samples, shown together with the histograms of parameter distribution in Figure 7. Such histograms provide a complete insight into the statistical properties of sample scattering. If estimates of *O* and *E* are given in terms of mean value and variance only, confidence intervals can be established, but they are affected by the presence of outliers (see e.g., Figure 7c) improperly estimated in the case of multimodal distributions (see e.g., Figure 7b), or in the case of heavily skewed distributions (see e.g., Figure 7e). One may instead adopt the mode of each posterior distribution as a more appropriate estimate, as it represents the value that appears most often in it. Accordingly, the estimated values $O_{m}$ and $E_{m}$, along with the relevant standard deviations are collected in Table 3 for the nine devices. The reported results show that overetch varies in the interval (−0.122 μm −0.020 μm), while the Young’s modulus varies in the interval (135.06 GPa 158.65 GPa).

**Figure 8:**
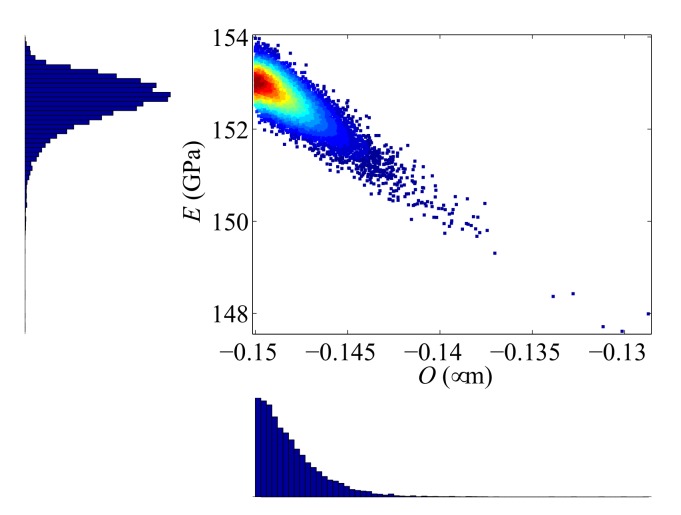
Scattering of the TMCMC samples, and corresponding histograms of the model parameter distributions for device #10.

Figure 8 finally collects the results corresponding to the mentioned device for which the parameter estimation process did not provide successful outcomes. The TMCMC samples are concentrated at the border of the considered domain of variation, which had been previously set in compliance with the features of the micro-fabrication process. Therefore, this particular sample distribution is assumed to be unrealistic due to some peculiar defects probably featured by the device. Even if the full-order model is supposed to properly simulate the electro-mechanical response of the device, the considered unknown parameters may not completely describe the micromechanically-governed imperfections for the present case; for instance, an initial offset of the rotor may have been induced by gradients of the residual stresses in the polysilicon film.

Although improvements of the FE model, to allow for additional sources of uncertainty at the microscale, can lead to better parameter estimation results, the framework here proposed, adopting a hybrid model order reduction technique and TMCMC simulations, has clearly shown its capabilities at accommodating detailed numerical models and Bayesian inverse analysis for parameter estimation at the microscale.

## 6. Conclusions

In this paper, we have proposed a fully stochastic approach to estimate microscale uncertainties linked to the polycrystalline morphology of the columnar film constituting the movable structures of inertial MEMS, and to the etching phase of the relevant micro-fabrication process. Estimates of the overetch depth *O* (assumed to be isotropic and homogeneous in the plane parallel to the substrate surface) and of the overall film stiffness (ruled by its Young’s modulus *E*) were obtained through a transitional Markov chain Monte Carlo approach, so as to get full insights into the probability distributions of the said parameters. By this approach, the effects of the (rather limited) measurement noise were also accounted for.

Ten devices, all supposed to be nominally identical, were experimentally tested to collect information on the signature of the aforementioned uncertain parameters on the input–output characteristics of the devices. By means of a standard electrostatic actuation, the responses, in terms of the capacitance change *C* induced by an applied voltage *V*, were obtained to feed the TMCMC analysis. As a term of comparison for the experimental data, a high fidelity finite element model, parametrized by the values of *O* and *E*, was adopted; within such a model, the nonlinear effects induced by electrostatic loading and by the mechanical deformation were fully accounted for.

To speed up the procedure, the mentioned high fidelity solutions were adopted to build a reduced-order model guaranteeing the required accuracy to obtain robust estimations of the unknowns. First, the *C*–*V* curves were processed via proper orthogonal decomposition to identify the fundamental modes governing the nonlinear system response, independently of the values of *O* and *E*. Next, this set of data was interpolated in the domain of variation of the parameters through kriging; specifically, the Clenshaw–Curtis sparse grid sampling method was used to address the deployment of the high fidelity analyses in the parameters’ space.

Results of this hybrid TMCMC/POD-kriging approach have shown that the ten samples, even if taken from the same wafer and even if nominally identical, were characterized by a response that is clearly governed by different values of *O* and *E*, not only in terms of mean (or mode) values, but also in terms of potential scattering around them.

Out of the ten devices here analyzed, the proposed procedure led to unsatisfactory outcomes only in one case, for which the estimates were considered too close to the boundary of their physically-sound domain of variation. This issue will be further considered in future investigations, wherein the current numerical model is going to be enhanced by allowing for additional uncertainty parameters governing the mechanical response of the polysilicon film (like e.g., anchor compliance) and the actual geometry of the movable parts (like e.g., either anisotropic or non-homogeneous overetch depths). 

## Figures and Tables

**Table 1:** Target geometric dimensions of the device (see Figure 1).

Parameter	Value
beam length	20 μm
beam width	2 μm
out-of-plane beam thickness	22 μm
initial gap at capacitors	2 μm
conductor length	83 μm
plate side length	200 μm

**Table 2:** Clenshaw–Curtis sparse grid sampling: dependence on the interpolation degree *d* of the number of FE analyses to run to build the POD-Kriging model, in case of a two-dimensional problem.

*d*	0	1	2	3	4	5
FE analyses	1	5	13	29	65	145

**Table 3:** Specimen-dependent estimated values of mode and standard deviation of the unknown model parameters *O* and *E*.

Specimen #	$O_{m}$ (μm)	$O_{\mathrm{std}}$ (μm)	$E_{m}$ (GPa)	$E_{\mathrm{std}}$ (GPa)
1	−0.106	0.0035	146.30	0.703
2	−0.092	0.0097	153.28	1.870
3	−0.095	0.0035	153.49	0.713
4	−0.102	0.0030	153.50	0.637
5	−0.122	0.0038	152.96	0.896
6	−0.053	0.0067	158.65	1.069
7	−0.067	0.0063	135.06	1.010
8	−0.020	0.0074	153.49	0.977
9	−0.020	0.0077	145.68	0.927

## Appendix A. Transitional Markov Chain Monte Carlo Algorithm

In Section 4, the TMCMC algorithm, as developed in [63], has been discussed as far as the sampling of the posterior PDF for parameter estimation purposes is concerned.

In this Appendix, we provide some additional technical details of the numerical procedure. At each iteration, the algorithmic steps can be summarized as follows:

1. The first guess for the intermediate PDF $p_{0}\left(x\right)$ is defined as the prior of parameters p(x|M). It is assumed in a form that allows sampling to obtain $x_{k}^{\left(0\right)}$, $k=1,\ldots ,N_{s}$.
2. The values of the tempering parameters are initially chosen such that the coefficient of variation for $w\left(x_{k}^{\left(0\right)}\right)=p{\left(C_{exp}|x_{k}^{\left(0\right)},M\right)}^{\eta_{1}}$, $k=1,\ldots ,N_{s}$, attains a prescribed tolerance allowing elitism diversification (see [48]).
3. Samples, $x_{k}^{\left(1\right)},k=1,\ldots ,N_{s}$, are generated adopting the Metropolis–Hastings algorithm. The *k*-th sample is drawn from a Markov chain that starts from a so-called sample leader, which is equal to one of the samples $x_{l}^{\left(0\right)}$, $l=1,\ldots ,N_{s}$; the probability of the leader to be $x_{l}^{\left(0\right)}$ is given by the normalized weight $\tilde{w}\left(x_{l}^{\left(0\right)}\right)$. The algorithm adopts a Gaussian proposal PDF centered at the current sample in the *l*-th chain, featuring a covariance matrix $\Sigma_{m}$ given by:
  (A1) $$\Sigma_{m}=\sum_{l=1}^{N_{s}}\tilde{w}\left(x_{l}^{\left(0\right)}\right)\left(x_{l}^{\left(0\right)}-{\bar{x}}^{\left(0\right)}\right){\left(x_{l}^{\left(0\right)}-{\bar{x}}^{\left(0\right)}\right)}^{T},$$
  where:
  (A2) $${\bar{x}}^{\left(0\right)}=\sum_{k=1}^{N_{s}}\tilde{w}\left(x_{k}^{\left(0\right)}\right)x_{k}^{\left(0\right)}.$$
  A scaling parameter can be adopted for $\Sigma_{m}$, in order to suppresses the rejection rate while dealing with large MCMC jumps (see [63]).
4. Steps (1) to (3) are repeated until $\eta_{\Gamma }=1$, and the available data are fully plugged into the posterior distribution update.
